# Albuminuria but not low eGFR is closely associated with atherosclerosis in patients with type 2 diabetes: an observational study

**DOI:** 10.1186/s13098-022-00824-x

**Published:** 2022-04-12

**Authors:** Jun-Wei Wang, Jiang-Feng Ke, Zhi-Hui Zhang, Jun-Xi Lu, Lian-Xi Li

**Affiliations:** Department of Endocrinology and Metabolism, Shanghai Clinical Center for Diabetes, Shanghai Diabetes Institute, Shanghai Key Laboratory of Diabetes Mellitus, Shanghai Key Clinical Center for Metabolic Disease, Shanghai Jiao Tong University Affiliated Sixth People’s Hospital, 600 Yishan Road, Shanghai, 200233 China

**Keywords:** Albuminuria, eGFR, Atherosclerosis, Type 2 diabetes

## Abstract

**Background:**

There is still controversy regarding the associations of urinary albumin excretion (UAE) and estimated glomerular filtration rate (eGFR) with atherosclerosis in patients with type 2 diabetes mellitus (T2DM). Therefore, it is necessary to explore the correlation between them in T2DM patients.

**Methods:**

We conducted a survey involving 2565 T2DM patients from a single center. The study cohort was classified into three groups based on the levels of albuminuria: normal UAE (UAE < 30 mg/24 h), moderate UAE (UAE between 30 and 299 mg/24 h) and high UAE (UAE ≥ 300 mg/24 h). Additionally, the patients were divided into three separate groups according to eGFR levels, including low eGFR (eGFR < 60 ml/min/1.73 m^2^), intermediate eGFR (eGFR 60–89 ml/min/1.73 m^2^) and normal eGFR (eGFR ≥ 90 ml/min/1.73 m^2^) groups. Atherosclerotic lesions were compared among the three UAE and eGFR groups. Regression analyses were used to assess the associations of atherosclerotic lesions with UAE and eGFR in T2DM.

**Results:**

After controlling for age, sex and diabetes duration, the prevalence of atherosclerotic plaque and stenosis were significantly increased from the normal to high UAE groups (plaque: 72.2%, 78.6% and 87.3%, respectively, p = 0.016 for trend; stenosis: 14.0%, 25.5% and 37.3%, respectively, p < 0.001 for trend). Likewise, the values of carotid intima-media thickness (CIMT) and femoral intima-media thickness (FIMT) were also obviously increased from the normal to high UAE groups (CIMT: p < 0.001 for trend; FIMT: p = 0.001 for trend). Conversely, only the FIMT value was clearly increased from the low to normal eGFR groups (p = 0.001 for trend). Fully adjusted regression analyses revealed that UAE was closely associated with the presence of atherosclerotic plaque (OR 1.20, 95% CI 1.03–1.40, p = 0.020) and stenosis (OR 1.17, 95% CI 1.01–1.35, p = 0.036), and with the values of CIMT (β 0.05, 95% CI 0.01–0.10, p = 0.029) and FIMT (β 0.07, 95% CI 0.03–0.11, p = 0.001) in T2DM patients. However, there was no significant association between eGFR levels and atherosclerotic lesions in T2DM after adjustment for multiple confounding factors.

**Conclusions:**

Overall, albuminuria rather than low eGFR is closely associated with atherosclerotic lesions in T2DM patients. Albuminuria is an independent risk factor for carotid and femoral atherosclerotic lesions in T2DM. Therefore, albuminuria may be a potential early marker to predict the development of atherosclerosis in patients with T2DM.

## Introduction

A high prevalence of atherosclerosis was revealed in patients with type 2 diabetes mellitus (T2DM). T2DM patients without underlying cardiovascular disease (CVD) have a high likelihood of developing carotid plaque, with a prevalence of 43–64% [1]. Likewise, atherosclerosis in the lower extremity artery was also observed in 21.2–72.9% of T2DM patients, which was a major hallmark of atherosclerosis [2–4]. Furthermore, the prevalence of cardiovascular events remained high in diabetic patients with atherosclerosis [5–7]. Therefore, early identification and intervention of atherosclerosis-related risk factors will be beneficial in reducing the occurrence of macrovascular events such as stroke and myocardial infarction in T2DM subjects [8, 9].

Currently, the risk of developing atherosclerosis and CVD is significantly high in T2DM patients with obesity, dyslipidemia, and hypertension, among others [10–12]. However, the correlation between atherosclerosis and kidney dysfunction manifested by increased albuminuria and/or decreased eGFR is probably not very clear in general and in T2DM populations. For example, in two previous studies, euglycemic individuals with albuminuria levels in the upper normal ranges had elevated intima-media thickness (IMT) and increased carotid plaque number [13, 14]. However, other studies did not find an association of microalbuminuria with IMT in the absence of diabetes and hypertension [15, 16]. Likewise, other studies, including our previous study, also found that high normal albuminuria was significantly associated with an increased risk of atherosclerotic lesions in T2DM patients [17–20]. However, there are other studies showing that albuminuria is not a predictor of atherosclerosis in carotid and peripheral arteries in T2DM patients [21, 22]. Therefore, the association between albuminuria and atherosclerosis in T2DM is possibly inconclusive and may be influenced by other risk factors.

On the other hand, the association between low eGFR and the risk of atherosclerosis still needs to be determined in general and in T2DM populations. Two previous investigations demonstrated that moderately to severely decreased eGFR was associated with carotid plaque and peripheral arterial disease (PAD) in the whole population [23, 24]. However, it has also been suggested that no statistically significant association was observed between eGFR and CIMT in the general population [25]. In addition, low eGFR has also been linked to peripheral atherosclerosis in patients with T2DM [22, 26]. However, in two separate studies, eGFR was not associated with atherosclerosis in T2DM patients [27, 28]. To date, there may be no clear evidence as to whether low eGFR is an accurate predictor of atherosclerosis in patients with T2DM.

Therefore, the associations of eGFR and albuminuria with atherosclerosis may be unclear in patients with diabetes [17–22, 26–31]. Furthermore, there are few studies investigating the correlation between eGFR/albuminuria and atherosclerosis in T2DM. Therefore, the aim of this study was to investigate the associations of albuminuria and low eGFR with atherosclerosis, including carotid and lower extremity atherosclerotic lesions, in Chinese patients with T2DM.

## Materials and methods

### Subjects and study design

Using a cross-sectional study design, T2DM patients hospitalized in the Department of Endocrinology and Metabolism, Shanghai Jiao Tong University Affiliated Sixth People’s Hospital, from January 2007 to June 2009 were enrolled in this study. The protocol for this study was approved by the ethics review boards of the same hospital. All study participants consented to the study in writing. The study was conducted in accordance with the Helsinki Declaration. It also complied with the STROBE guidelines. It is worth noting that some of the data included in this report were extracted from our previous study, such as the history of hypertension, duration of diabetes (DD), smoking and alcohol status, and medicine use, including antihypertensive agents (AHA), lipid-lowering drugs (LLD), antiplatelet agents (APA), metformin, insulin sensitizers, and insulin or insulin analogue (IIA) [11]. Smoking and alcohol status were defined in our previous studies [4, 7, 11]. Specifically, smoking included both current and former smokers. Similarly, alcohol use included current and former use of alcohol.

The inclusion criteria were as follows: patients with a clear previous history of T2DM or patients diagnosed with T2DM according to the 1999 World Health Organization (WHO) criteria during hospitalization [32] and patients aged greater than or equal to 17 years old. Patients without complete data and those below 17 years old were excluded from the study. In the end, 2565 patients meeting the inclusion criteria were included in the subsequent analyses. The patients were divided into three groups based on either albuminuria or eGFR levels: normal UAE (< 30 mg/24 h), moderate UAE (30–299 mg/24 h) and high UAE (≥ 300 mg/24 h) groups and low eGFR (< 60 ml/min/1.73 m^2^), intermediate eGFR (60–89 ml/min/1.73 m^2^) and normal eGFR (≥ 90 ml/min/1.73 m^2^) groups. The eGFR grouping in the present study was based on the latest definition of CKD [33, 34].

To further elucidate the relationship between atherosclerosis, UAE and eGFR, the patients were further classified into four groups: normal group (eGFR ≥ 90 ml/min/1.73 m^2^ and UAE < 30 mg/24 h), normal eGFR and high UAE group (eGFR ≥ 90 ml/min/1.73 m^2^ and UAE ≥ 30 mg/24 h), low eGFR and normal UAE group (eGFR < 90 ml/min/1.73 m^2^ and UAE < 30 mg/24 h), and low eGFR and high UAE group (eGFR < 90 ml/min/1.73 m^2^ and UAE ≥ 30 mg/24 h).

### Physical examination and laboratory tests

The height, waist and hip circumferences, weight and blood pressure of the participants were measured as previously described at the time of admission [4, 35, 36]. Body mass index (BMI) was calculated as the weight divided by the square of the height. The waist-to-hip ratio (WHR) was measured as the ratio of waist circumference to hip circumference. Blood samples were taken after an overnight fast and two hours after breakfast on the second day after enrollment. Blood glucose levels, lipid profiles, islet function, and liver and renal functions were measured as previously described [4, 35, 36]. Serum creatinine levels were determined by the sarcosine oxidase method. Insulin resistance assessment was performed using the homeostasis assessment of insulin resistance model (accessed at http://www.dtu.ox.ac.uk). The 24-h UAE was determined based on the mean value of three 24-h UAEs after admission. Albuminuria was measured by immune scattering turbidimetry. The eGFR was calculated based on the equation recommended for the Chinese population [175×(serum creatinine)^−1.234^× (age)^−0.179^(×0.79, if female)] [11].

### Ultrasonography tests

Ultrasonography of the carotid and lower extremity arteries, including measurements for CIMT, FIMT, atherosclerotic plaque and stenosis, was performed as previously described [4, 35, 37]. Specifically, ultrasound examinations were performed by three echographers independently using an Acuson Sequoia 512 machine with a probe frequency of 5–13 MHz [4]. In this study, CIMT, FIMT, plaque and stenosis were defined as previously described [4, 7, 35, 37]. In brief, IMT was defined as the length between the leading edge of the lumen-intima echo and the leading edge of the media-adventitia echo. Atherosclerotic plaque was regarded as a lesion that invaded the lumen of an artery by 0.5 mm or by half the surrounding IMT value, or an IMT of more than 1.5 mm in any arterial segment. Additionally, stenosis was regarded as the stenosis of either the carotid or femoral arteries [4, 7, 35, 37].

### Statistical analysis

Data were analyzed using SPSS 15.0 (SPSS Inc., Chicago, IL, USA). Continuous variables were assessed for normality and thereafter expressed as the mean ± standard deviation or the median and quartile range. Differences among three groups were analyzed using one-way ANOVA or the Kruskal–Wallis test. Specifically, normally distributed variables were analyzed using one-way ANOVA, and nonnormally distributed variables were measured using the Kruskal–Wallis test. Categorical variables were analyzed using chi-square tests. The relationship between categorical variables and parameters of interest while controlling for sex and/or age was analyzed using logistic regression. Differences in quantitative variables while adjusting for sex and/or age were assessed using univariate linear regression models. The associations of UAE with atherosclerotic plaque/stenosis were estimated using binary logistic regression. Likewise, the associations between eGFR and atherosclerotic plaque/stenosis were also analyzed by a binary logistic regression model. Furthermore, the association of IMT with UAE and eGFR in patients with diabetes was calculated by univariate linear regression. Additionally, nonnormally distributed variables were transformed by normal score transformation before entering into regression analyses. We constructed five models to assess the relationship between atherosclerotic lesions and either UAE or eGFR. For Model 1, there were no adjustments for any variables. In Model 2, there were adjustments for age, sex, DD, hypertension, smoking status and alcohol use. Model 3 incorporated additional adjustments for AHA, LLD, and APA use. In Model 4, there were additional adjustments for systolic blood pressure (SBP), diastolic blood pressure (DBP), BMI, waist circumference (WC) and WHR. Model 5 had the greatest adjustment, further incorporating alanine transaminase (ALT), total triglycerides (TG), total cholesterol (TC), high-density lipoprotein cholesterol (HDL-C), low-density lipoprotein cholesterol (LDL-C), serum uric acid (SUA), fasting plasma glucose (FPG), 2-h postprandial plasma glucose (2-h PPG), glycated hemoglobin A1c (HbA1c), fasting C-peptide (FCP), 2-h postprandial C-peptide (2-h PCP) and HOMA of insulin resistance (HOMA2-IR) to the previously mentioned variables. Statistical significance was set at p < 0.05.

## Results

### Characteristics of the study subjects

The baseline characteristics of the subjects grouped by either UAE or eGFR levels are highlighted in Tables 1 and 2, respectively. As shown in Table 1, after adjustment for sex and age, the prevalence of hypertension, the percentage of IIA and LLD and AHA use, SBP, DBP, WHR, FCP, HOMA2-IR, TG, TC, and SUA were found to increase with increasing UAE (all p < 0.05). Additionally, there were significant differences in the percentage of insulin sensitizers use, WC, BMI, FPG, HDL-C, and LDL-C among the three UAE groups in T2DM patients (all p < 0.05). However, there was no significant difference in the percentage of drinking, smoking, metformin and APA use, 2-h PPG, HbA1c, 2-h CP, or ALT among the three groups.

**Table 1:** Characteristics of the subjects according to UAE levels

Variables	UAE < 30 mg/24 h (n = 1940)	UAE 30–299 mg/24 h (n = 467)	UAE ≥ 300 mg/24 h (n = 158)	p value	p* value
Male (n, %)	1066 (54.9%)	253 (54.2%)	109 (69.0%)	0.002	< 0.001
Age (years)	59 ± 12	61 ± 13	61 ± 12	< 0.001	< 0.001
DD (months)*	72 (17–120)	108 (36–156)	120 (48–180)	< 0.001	< 0.001
Hypertension (n, %)	931 (48.0%)	316 (67.7%)	129 (81.6%)	< 0.001	< 0.001
Drinking (n, %)	298 (15.4%)	73 (15.6%)	26 (16.5%)	0.930	0.688
Smoking (n, %)	545 (28.1%)	139 (29.8%)	54 (34.2%)	0.233	0.204
APA (n, %)	981 (50.6%)	244 (52.2%)	92 (58.2%)	0.164	0.601
LLD (n, %)	560 (28.9%)	163 (34.9%)	76 (48.1%)	< 0.001	< 0.001
AHA (n, %)	824 (42.5%)	307 (65.7%)	129 (81.6%)	< 0.001	< 0.001
IIA (n, %)	1333 (68.7%)	367 (78.6%)	146 (92.4%)	< 0.001	< 0.001
Metformin (n, %)	1087 (56.0%)	268 (57.4%)	89 (56.3%)	0.868	0.678
Insulin sensitizers (n, %)	264 (13.6%)	95(20.3%)	18(11.4%)	0.001	0.001
SBP (mmHg)	130 ± 16	137 ± 18	149 ± 20	< 0.001	< 0.001
DBP (mmHg)	79 ± 9	82 ± 11	85 ± 11	< 0.001	< 0.001
WC (cm)	88.41 ± 9.88	92.25 ± 11.01	91.76 ± 11.10	< 0.001	< 0.001
WHR	0.91 ± 0.06	0.92 ± 0.07	0.93 ± 0.06	< 0.001	< 0.001
BMI (kg/m^2^)	24.64 ± 3.33	25.7 ± 3.67	25.54 ± 3.59	< 0.001	< 0.001
FPG (mmol/l)*	7.72 (6.21–9.66)	8.1 (6.42–9.93)	7.56 (5.86–9.59)	0.045	0.006
2-h PPG (mmol/l)*	13.58 (10.24–16.98)	13.79 (10.56–17.18)	12.37 (9.1–16.34)	0.018	0.180
HbA1c (%)	9.11 ± 2.38	9.35 ± 2.27	9.12 ± 2.37	0.150	0.087
FCP (ng/mL)*	1.61 (0.98–2.34)	1.75 (1.04–2.62)	1.87 (1.045–3.01)	< 0.001	< 0.001
2-h PCP (ng/mL)*	3.67 (2.07–5.35)	3.51 (2.09–5.35)	3.6 (1.79–5.34)	0.854	0.814
HOMA2-IR*	1.4 (0.9-2)	1.5 (0.9–2.4)	1.6 (0.9–2.5)	0.001	< 0.001
TG (mmol/l)*	1.37 (0.96–2.04)	1.59 (1.14–2.39)	1.66 (1.21–2.49)	< 0.001	< 0.001
TC (mmol/l)	4.66 ± 1.04	4.74 ± 1.20	5.52 ± 1.47	< 0.001	< 0.001
HDL-C (mmol/l)	1.13 ± 0.31	1.06 ± 0.28	1.11 ± 0.30	< 0.001	< 0.001
LDL-C (mmol/l)	3.09 ± 0.92	3.09 ± 0.96	3.63 ± 1.21	< 0.001	< 0.001
ALT (U/l) *	19 (13–30)	20 (14–33)	18 (12–26)	0.049	0.103
SUA (µmol/l) *	302 (251–364)	320 (262–391)	378 (304–450)	< 0.001	< 0.001

Values are expressed as the mean ± S.D, or median with interquartile range, or percentages

*UAE* urinary albumin excretion, *DD* duration of diabetes, *APA* anti-platelet agents, *LLD* lipid-lowering drugs, *AHA* anti-hypertensive agents, *IIA* insulin or insulin analogue, *SBP* systolic blood pressure, *DBP* diastolic blood pressure, *WC* waist circumference, *WHR* waist-to-hip ratio, *BMI* body mass index, *FPG* fasting plasma glucose, *2-h PPG* 2-h postprandial plasma glucose, *HbA1c* glycated hemoglobin A1c, *FCP* fasting C-peptide, *2-h PCP* 2-h postprandial C-peptide, *HOMA2-IR* HOMA of insulin resistance, *TG* total triglycerides *TC* total cholesterol, *HDL-C* high-density lipoprotein cholesterol, *LDL-C* low-density lipoprotein cholesterol, *ALT* alanine transaminase, *SUA* serum uric acid

p value: The p-values were not adjusted for age and sex for the trend

p* value: The p-values were adjusted for sex and age for the trend

* The Kruskal-Wallis test was applied

**Table 2:** Characteristics of the subjects according to eGFR levels

Variables	eGFR ≥ 90ml/min/1.73m^2^ (n = 1684)	eGFR 60–89 ml/min/1.73m^2^ (n = 719)	eGFR < 60ml/min/1.73m^2^ (n = 162)	p value	p* value
Male (n, %)	930 (55.2%)	410 (57.0%)	88 (54.3%)	0.674	< 0.001
Age (years)	56 ± 12	66 ± 10	69 ± 10	< 0.001	< 0.001
DD (months)*	72 (12–120)	96 (36–156)	120 (60–192)	< 0.001	0.234
Hypertension (n, %)	788 (46.8%)	456 (63.4%)	132 (81.5%)	< 0.001	< 0.001
Drinking (n, %)	287 (17.0%)	100 (13.9%)	10 (6.2%)	< 0.001	0.025
Smoking (n, %)	520 (30.9%)	185 (25.7%)	33 (20.4%)	0.002	0.358
APA (n, %)	789 (46.9%)	436 (60.6%)	92 (56.8%)	< 0.001	0.066
LLD (n, %)	509 (30.2%)	234 (32.5%)	56 (34.6%)	0.332	0.093
AHA (n, %)	700 (41.6%)	434 (60.4%)	126 (77.8%)	< 0.001	< 0.001
IIA (n, %)	1209 (71.8%)	491 (68.3%)	146 (90.1%)	< 0.001	< 0.001
Metformin (n, %)	993 (59.0%)	378 (52.6%)	73 (45.1%)	< 0.001	0.016
Insulin sensitizers (n,%)	258 (15.3%)	97 (13.5%)	22 (13.6%)	0.464	0.642
SBP (mmHg)	130 ± 17	134 ± 18	141 ± 21	< 0.001	< 0.001
DBP (mmHg)	80 ± 10	80 ± 10	80 ± 11	< 0.001	0.918
WC (cm)	88.62 ± 10.24	90.25 ± 10.32	92.51 ± 9.67	< 0.001	< 0.001
WHR	0.91 ± 0.06	0.91 ± 0.07	0.93 ± 0.06	< 0.001	0.009
BMI (kg/m^2^)	24.7 ± 3.48	25.13 ± 3.21	25.74 ± 3.69	< 0.001	< 0.001
FPG (mmol/l)*	8.07 (6.48–10.04)	7.27 (5.9-9)	7.06 (5.62–8.89)	< 0.001	< 0.001
2-h PPG (mmol/l)*	13.81 (10.51–17.17)	13.14 (9.66–16.49)	12.15 (9.58–16.94)	0.005	0.137
HbA1c (%)	9.34 ± 2.34	8.79 ± 2.29	8.81 ± 2.69	0.150	< 0.001
FCP (ng/mL)*	1.58 (0.94–2.33)	1.7 (1.1–2.53)	2.21 (1.2–3.58)	< 0.001	< 0.001
2-h PCP (ng/mL)*	3.46 (1.88–5.18)	4.12 (2.43–5.53)	4.18 (2.32–5.82)	< 0.001	< 0.001
HOMA2-IR*	1.4 (0.8-2)	1.4 (0.9–2.1)	2 (0.9–2.9)	0.050	< 0.001
TG (mmol/l)*	1.43 (0.99–2.14)	1.42 (1–2.03)	1.55 (1.08–2.39)	0.512	< 0.001
TC (mmol/l)	4.73 ± 1.1	4.67 ± 1.08	4.97 ± 1.45	< 0.001	0.001
HDL-C (mmol/l)	1.12 ± 0.3	1.11 ± 0.3	1.06 ± 0.3	< 0.001	< 0.001
LDL-C (mmol/l)	3.14 ± 0.95	3.07 ± 0.93	3.2 ± 1.08	< 0.001	0.251
ALT (U/l)*	20 (14–31)	19 (13–27)	16 (12–26)	0.006	0.607
SUA (µmol/l) *	292 (244–351)	330 (278–391)	414 (349–495)	< 0.001	< 0.001

Values are expressed as the mean ± S.D, or median with interquartile range, or percentages

*eGFR* estimated glomerular filtration rate, *DD* duration of diabetes, *APA* anti-platelet agents, *LLD* lipid-lowering drugs, *AHA* anti-hypertensive agents, *IIA* insulin or insulin analogue, *SBP* systolic blood pressure, *DBP* diastolic blood pressure, *WC* waist circumference, *WHR* waist-to-hip ratio, *BMI* body mass index, *FPG* fasting plasma glucose, *2-h PPG* 2-h postprandial plasma glucose, *HbA1c* glycated hemoglobin A1c, *FCP* fasting C-peptide, *2-h PCP* 2-h postprandial C-peptide, *HOMA2-IR* HOMA of insulin resistance, *TG* total triglycerides, *TC* total cholesterol, *HDL-C* high-density lipoprotein cholesterol, *LDL-C* low-density lipoprotein cholesterol, *ALT* alanine transaminase, *SUA* serum uric acid

p value: The p-values were not adjusted for sex and age for the trend

p* value: The p-values were adjusted for sex and age for the trend

* The Kruskal-Wallis test was applied

Table 2 shows the baseline characteristics of the different eGFR groups. After adjustment for age and sex, lower eGFR was associated with older age, longer DD, higher prevalence of hypertension, more AHA usage, less metformin usage, and higher SBP, WC, BMI, FCP, 2-h-PCP, HOMA2-IR, and SUA but lower HDL-C (all p < 0.05). Moreover, the percentage of drinking and smoking, APA and IIA usage, sex, WHR, HbA1C, TG, TC, and FPG varied among the different eGFR groups (all p < 0.05), whereas insulin sensitizers and LLD use, DBP, LDL-C, 2-h-PPG, and ALT were indiscriminate (all p > 0.05).

### Comparison of atherosclerotic lesions among different groups

**Fig. 1:**
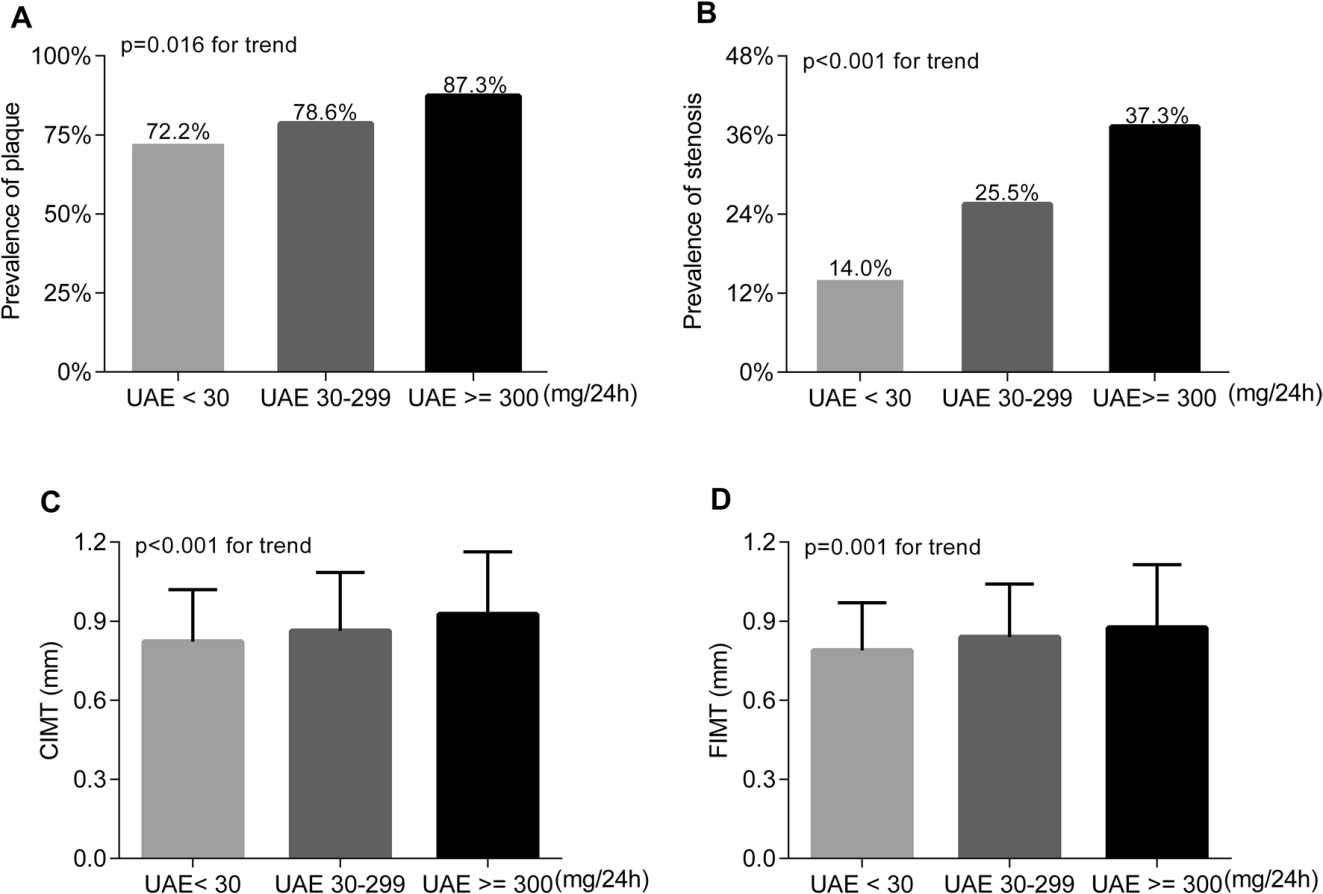
Comparison of atherosclerotic lesions among the UAE groups. **A** Comparison of the prevalence of atherosclerotic plaque among the UAE groups after adjusting for age, sex, and DD. **B** Comparison of the prevalence of atherosclerotic stenosis among the UAE groups after adjustment for age, sex, and DD. **C** Comparison of the CIMT values among the UAE groups after controlling for age, sex, and DD. **D** Comparison of the FIMT values among the UAE groups after adjusting for age, sex, and DD

Figure 1 compares the atherosclerotic lesions among the different UAE groups after adjustment for age, sex, and DD. The prevalence of plaques (72.2% vs. 78.6% and 87.3%; p = 0.016 for trend) and stenosis (14.0% vs. 25.5% and 37.3%; p < 0.001 for trend) was significantly increased from the normal to high UAE groups in T2DM (Fig. 1 A and 1B). Likewise, both the CIMT (0.82 ± 0.20, 0.86 ± 0.22 and 0.93 ± 0.24 mm, p < 0.001 for trend) and FIMT (0.79 ± 0.18, 0.84 ± 0.20 and 0.88 ± 0.24 mm, p = 0.001 for trend) values also gradually increased from normal to high UAE levels (Fig. 1 C and 1D).

**Fig. 2:**
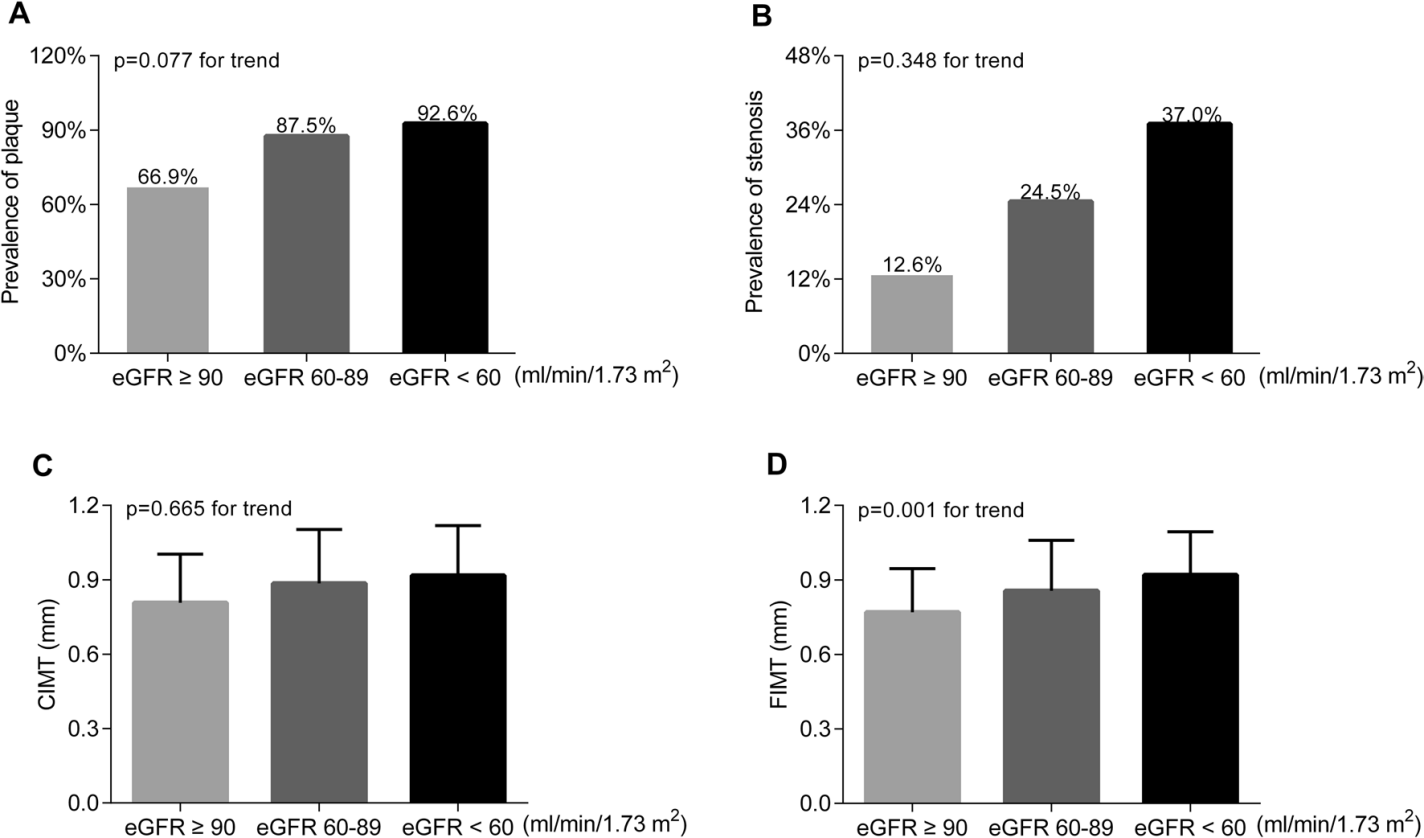
Comparison of atherosclerotic lesions according to eGFR status. **A** Comparison of the prevalence of plaque among the eGFR groups after adjusting for age, sex, and DD. **B** Comparison of the prevalence of stenosis among the eGFR groups after adjustment for age, sex, and DD.** C** Comparison of the CIMT values among the eGFR groups after controlling for age, sex, and DD.** D** Comparison of the FIMT values among the eGFR groups after adjusting for age, sex, and DD

However, after controlling for age, sex, and DD, only the FIMT (0.92 ± 0.17, 0.86 ± 0.20 and 0.77 ± 0.18 mm, p = 0.001 for trend) value was significantly different from those of the low to normal eGFR groups (Fig. 2D). There was no significant difference in the prevalence of atherosclerotic plaques (92.6% vs. 87.5% and 66.9%; p = 0.077 for trend) and stenosis (37.0% vs. 24.5% and 12.6%; p = 0.348 for trend) or in the CIMT value (0.92 ± 0.20, 0.89 ± 0.22 and 0.81 ± 0.20 mm, p = 0.665 for trend) between the low and normal eGFR groups (Fig. 2A–C).

**Fig. 3:**
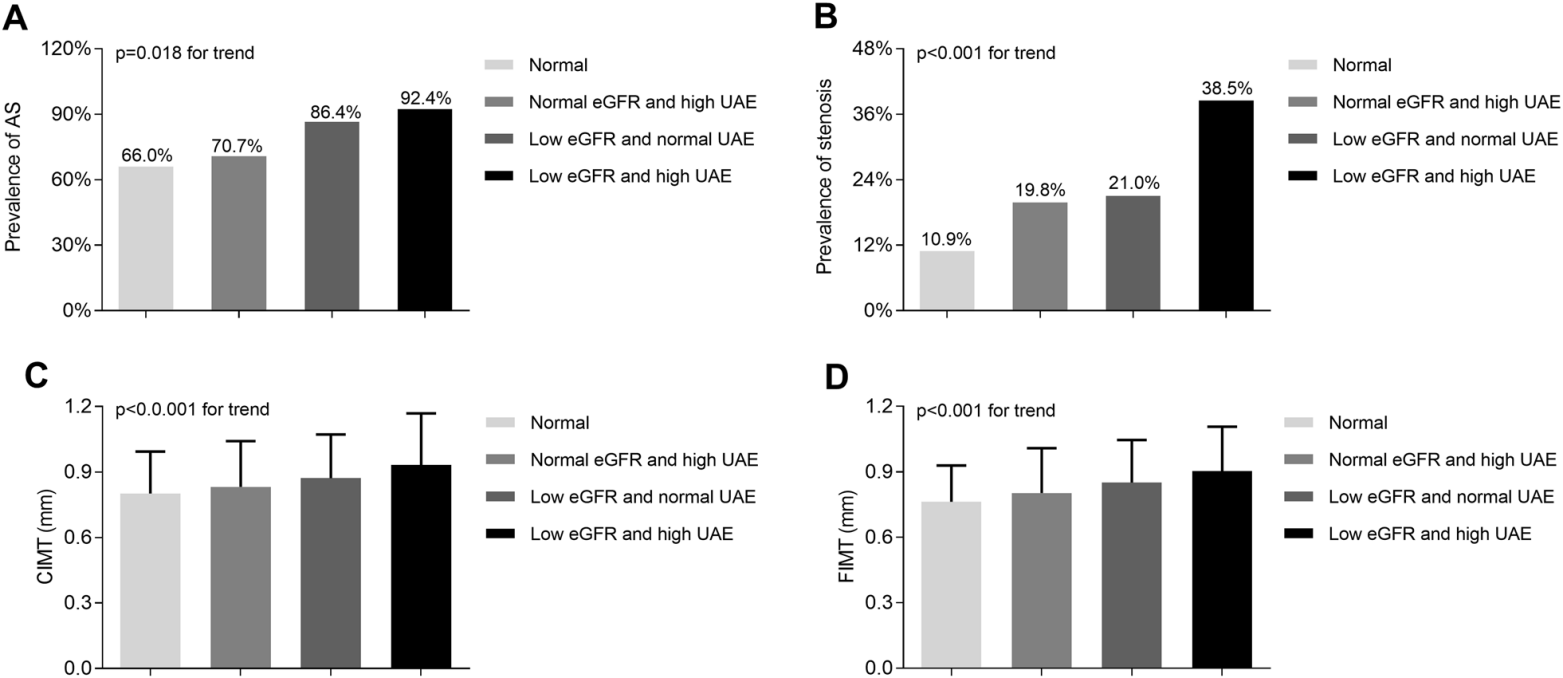
Comparison of atherosclerotic lesions among normal group, normal eGFR and high UAE group, low eGFR and normal UAE group and combined low eGFR and high UAE group. **A** Comparison of the prevalence of plaque among the four groups after adjusting for age, sex, and DD.** B** Comparison of the prevalence of stenosis among the four groups after adjustment for age, sex, and DD.** C** Comparison of the CIMT values among the four groups after controlling for age, sex, and DD. **D** Comparison of the FIMT values among the four groups after adjusting for age, sex, and DD

The comparison of atherosclerotic lesions in normal, normal eGFR and high UAE, low eGFR and normal UAE, and combined low eGFR and high UAE groups is demonstrated in Figure 3. After controlling for age, sex, and DD, the prevalence of atherosclerosis was higher in combined low eGFR and high UAE group (92.4%) than that in low eGFR and normal UAE group (86.4%), normal eGFR and high UAE group (70.7%), and normal group (66.0%) (p = 0.018 for trend) (Fig. 3A). Similarly, the prevalence of stenosis in combined low eGFR and high UAE group (38.5%) was higher than that in low eGFR and normal UAE group (21.0%), normal eGFR and high UAE group (19.8%), and normal group (10.9%) with a p value < 0.001 for trend (Fig. 3B). Moreover, levels of CIMT and FIMT tended to increase in patients with coexisting high UAE and low eGFR than in patients with normal UAE and eGFR, normal eGFR and high UAE, and low eGFR and normal UAE (p < 0.001 for trend) (Fig. 3C and 3D).

### The association of atherosclerotic plaque and stenosis with UAE and eGFR

Table 3 shows multiple regression analyses for the association of UAE and eGFR with atherosclerotic plaque and stenosis in T2DM. High UAE increased the risk of developing plaque and stenosis (OR 1.22, 95% CI 1.11 to 1.35, p < 0.001 for plaque; OR: 1.30, 95% CI 1.17 to 1.45, p < 0.001 for stenosis) without adjustments for variables (Model 1). After adjustment for age, sex, smoking, drinking, duration of diabetes and hypertension (Model 2), high UAE still increased the risk of developing plaque and stenosis (OR 1.16, 95% CI 1.03 to 1.30, p = 0.017 for plaque; OR 1.27, 95% CI 1.13 to 1.43, p < 0.001 for stenosis). In addition, after further adjustment for the use of AHA, LLD, and APA (Model 3) and SBP, DBP, BMI, WC and WHR (Model 4), high UAE still retained an independent relevance to the presence of plaque and stenosis (Model 3: OR 1.19, 95% CI 1.05 to 1.35, p = 0.007 for plaque; OR: 1.26, 95% CI 1.11 to 1.42, p < 0.001 for stenosis; Model 4: OR 1.15, 95% CI 1.00 to 1.32, p = 0.046 for plaque; OR 1.22, 95% CI 1.07 to 1.39, p = 0.003 for stenosis). Even after incorporation of additional clinical parameters (Model 5), high UAE was still closely associated with an increased risk of developing both plaque and stenosis (OR 1.20, 95% CI 1.03 to 1.39, p = 0.020; OR 1.17, 95% CI 1.01 to 1.35, p = 0.036, respectively). Before adjustment for variables, low eGFR was correlated with the prevalence of plaque and stenosis (OR: 0.49, 95% CI 0.44 to 0.54, p < 0.001; OR 0.61, 95% CI 0.54 to 0.68, p < 0.001, respectively). However, after further adjustment for selected variables, low eGFR was no longer associated with the risk of developing plaque and stenosis (Model 2: OR 0.90, 95% CI 0.79 to 1.02, p = 0.098 for plaque and OR 1.04, 95% CI 0.91 to 1.18, p = 0.614 for stenosis; Model 3:OR 0.88, 95% CI 0.77 to 1.01, p = 0.059 for plaque and OR 1.04, 95% CI 0.91 to 1.19, p = 0.593 for stenosis; Model 4:OR 0.88, 95% CI 0.76 to 1.02, p = 0.079 for plaque and OR 1.08, 95% CI 0.93 to 1.25, p = 0.312 for stenosis; Model 5:OR 0.88, 95% CI 0.75 to 1.02, p = 0.089 for plaque and OR 1.12, 95% CI 0.95 to 1.31, p = 0.177 for stenosis).

**Table 3:** Association of atherosclerotic plaques and stenosis with UAE and eGFR

	UAE		eGFR	
	OR (95% CI)	p values	OR (95% CI)	p values
Plaques				
Model 1	1.22 (1.11–1.35)	< 0.001	0.49 (0.44–0.54)	< 0.001
Model 2	1.16 (1.03–1.30)	0.017	0.90 (0.79–1.02)	0.098
Model 3	1.19 (1.05–1.35)	0.007	0.88 (0.77–1.01)	0.059
Model 4	1.15 (1.00–1.32)	0.046	0.88 (0.76–1.02)	0.079
Model 5	1.20 (1.03–1.39)	0.020	0.88 (0.75–1.02)	0.089
Stenosis				
Model 1	1.30 (1.17–1.45)	< 0.001	0.61 (0.54–0.68)	< 0.001
Model 2	1.27 (1.13–1.43)	< 0.001	1.04 (0.91–1.18)	0.614
Model 3	1.26 (1.11–1.42)	< 0.001	1.04 (0.91–1.19)	0.593
Model 4	1.22 (1.07–1.39)	0.003	1.08 (0.93–1.25)	0.312
Model 5	1.17 (1.01–1.35)	0.036	1.12 (0.95–1.31)	0.177

*UAE* urinary albumin excretion, *eGFR* estimated glomerular filtration rate, *DD* duration of diabetes, *APA* anti-platelet agents, *LLD* lipid-lowering drugs, *AHA* anti-hypertensive agents, *SBP* systolic blood pressure, *DBP* diastolic blood pressure, *WC* waist circumference, *WHR* waist-to-hip ratio, *BMI* body mass index, *FPG* fasting plasma glucose, *2-h PPG* 2-h postprandial plasma glucose, *HbA1c* glycated hemoglobin A1c, *FCP* fasting C-peptide, *2-h PCP* 2-h postprandial C-peptide, *HOMA2-IR* HOMA of insulin resistance, *TG* total triglycerides, *TC* total cholesterol, *HDL-C* high-density lipoprotein cholesterol, *LDL-C* low-density lipoprotein cholesterol, *ALT* alanine transaminase, *SUA* serum uric acid

Model 1: unadjusted

Model 2: Adjusted for age, sex, DD, hypertension, smoking status and alcohol use

Model 3: Further adjustment for use of AHA, LLD, APA

Model 4: Further adjustment for SBP, DBP, BMI, WC and WHR

Model 5: Further adjustment for ALT, TG, TC, HDL-C, LDL-C, SUA, FPG, 2-h PPG, HbA1c, FCP, 2-h PCP, HOMA2-IR

### The association of CIMT and FIMT with UAE and eGFR

As shown in Table 4, in unadjusted analysis, high UAE significantly impacted CIMT and FIMT values (β: 0.10, 95% CI 0.06 to 0.14, p < 0.001 for CIMT and β: 0.10, 95% CI 0.06 to 0.14, p < 0.001 for FIMT) (Model 1). After adjustment for age, sex, smoking, drinking, DD, and hypertension (Model 2), high UAE was correlated with increased CIMT (β: 0.07, 95% CI 0.04 to 0.11, p < 0.001) and FIMT (β: 0.08, 95% CI 0.04 to 0.11, p < 0.001) values. Moreover, after further controlling for the use of AHA, LLD, and APA (Model 3) and SBP, DBP, BMI, WC and WHR (Model 4), higher UAE remained significantly associated with thicker CIMT and FIMT (Model 3: β: 0.07, 95% CI 0.03 to 0.10, p < 0.001 for CIMT and β: 0.07, 95% CI 0.04 to 0.11, p < 0.001 for FIMT; Model 4: β: 0.05, 95% CI 0.02 to 0.09, p = 0.006 for CIMT and β: 0.08, 95% CI 0.04 to 0.12, p < 0.001 for FIMT). After adjustment for all variables (Model 5), regression analyses revealed that UAE was closely associated with CIMT and FIMT (β: 0.05, 95% CI 0.01 to 0.09, p = 0.029 for CIMT and β: 0.07, 95% CI 0.03 to 0.11, p = 0.001 for FIMT). However, low eGFR was associated with greater IMT in T2DM patients only in unadjusted analysis (β: − 0.23, 95% CI − 0.27 to − 0.20, p < 0.001 for CIMT and β: − 0.27, 95% CI − 0.31 to − 0.23, p < 0.001 for FIMT). There was no significant relationship between eGFR and CIMT/FIMT values after adjustment for clinical and biochemical parameters (Model 2: β: − 0.02, 95% CI − 0.06 to 0.03, p = 0.461 for CIMT and β: − 0.04, 95% CI − 0.08 to 0.00, p = 0.053 for FIMT; Model 3: β: − 0.01, 95% CI − 0.05 to 0.03, p = 0.486 for CIMT and β: − 0.04, 95% CI − 0.08 to 0.00, p = 0.052 for FIMT; Model 4: β: − 0.01, 95% CI − 0.05 to 0.03, p = 0.660 for CIMT and β: − 0.04, 95% CI − 0.08 to 0.00, p = 0.070 for FIMT; Model 5: β: − 0.01, 95% CI − 0.05 to 0.04, p = 0.701 for CIMT and β: − 0.04, 95% CI − 0.08 to 0.01, p = 0.134 for FIMT).

**Table 4:** Association of CIMT and FIMT with UAE and eGFR

	UAE		eGFR	
	β (95% CI)	p values	β (95% CI)	p values
CIMT				
Model 1	0.10 (0.06–0.14)	< 0.001	− 0.23 (-0.27∽-0.20)	< 0.001
Model 2	0.07 (0.04–0.11)	< 0.001	− 0.02 (-0.06–0.03)	0.461
Model 3	0.07 (0.03–0.10)	< 0.001	− 0.01 (-0.05–0.03)	0.486
Model 4	0.05 (0.02–0.09)	0.006	− 0.01 (-0.05–0.03)	0.660
Model 5	0.05 (0.01–0.09)	0.029	− 0.01 (-0.05–0.04)	0.701
FIMT				
Model 1	0.10 (0.06–0.14)	< 0.001	− 0.27 (-0.31∽-0.23)	< 0.001
Model 2	0.08 (0.04–0.11)	< 0.001	− 0.04 (-0.08–0.00)	0.053
Model 3	0.07 (0.04–0.11)	< 0.001	− 0.04 (-0.08–0.00)	0.052
Model 4	0.08 (0.04–0.12)	< 0.001	− 0.04 (-0.08–0.00)	0.070
Model 5	0.07 (0.03–0.11)	0.001	− 0.04 (-0.08–0.01)	0.134

*UAE* urinary albumin excretion, *eGFR* estimated glomerular filtration rate, *DD* duration of diabetes, *APA* anti-platelet agents, *LLD* lipid-lowering drugs, *AHA* anti-hypertensive agents, *SBP* systolic blood pressure, *DBP* diastolic blood pressure, *WC* waist circumference, *WHR* waist-to-hip ratio, *BMI* body mass index, *FPG* fasting plasma glucose, *2-h PPG* 2-h postprandial plasma glucose, *HbA1c* glycated hemoglobin A1c, *FCP* fasting C-peptide, *2-h PCP* 2-h postprandial C-peptide, *HOMA2*-*IR* HOMA of insulin resistance, *TG* total triglycerides, *TC* total cholesterol, *HDL*-*C* high-density lipoprotein cholesterol, *LDL-C* low-density lipoprotein cholesterol, *ALT* alanine transaminase, *SUA* serum uric acid

Model 1: unadjusted

Model 2: Adjusted for age, sex, DD, hypertension, smoking status and alcohol use

Model 3: Further adjustment for use of AHA, LLD, APA

Model 4: Further adjustment for SBP, DBP, BMI, WC and WHR

Model 5: Further adjustment for ALT, TG, TC, HDL-C, LDL-C, SUA, FPG, 2-h PPG, HbA1c, FCP, 2-h PCP, HOMA2-IR

## Discussion

It is well established that DM patients with either low eGFR or high albuminuria are at high risk of cardiovascular events [38–40]. An increased urine albumin-to-creatinine ratio (UACR) is found to predict the risk of cardiovascular events and major PAD in T2DM patients [38–40]. Likewise, several trials have shown that low eGFR is also associated with an increased risk of developing macrovascular diseases in patients with diabetes [39, 41, 42]. However, there are few studies exploring the associations of albuminuria and decreased eGFR with the development of atherosclerosis in T2DM subjects [17, 22, 27, 29–31]. Furthermore, the relationship between albuminuria or eGFR and atherosclerosis in patients with T2DM is controversial. Therefore, we performed the present study and demonstrated that high UAE, but not low eGFR, was an independent risk factor for atherosclerotic lesions in patients with T2DM.

The correlation between albuminuria and atherosclerosis is controversial in T2DM patients. In the general population, there was a dose–response relationship between albuminuria and the severity of carotid and femoral atherosclerosis in terms of IMT and atherosclerosis scores [43]. However, a previous study found that albuminuria was only associated with PAD but not with carotid plaque or CIMT in T2DM patients [21]. Moreover, Sjöblom et al. reported that UACR was not an independent predictive factor for CIMT after adjustment for blood pressure, HbA1c and LDL-C in T2DM patients [27]. Notably, albuminuria but not eGFR was clearly associated with atherosclerosis in T2DM patients in our study. There was a close relationship between UAE and the CIMT and FIMT values. Additionally, high UAE also increased the risk of atherosclerotic plaque and stenosis even after adjustment for clinical and biochemical parameters in our study. Consistent with our findings, Yokoyama et al. and Yamashita et al. found that an elevation in albuminuria was an important determinant of increased CIMT in T2DM patients[22, 44]. Likewise, Nomura et al. also found a significant difference in the prevalence of carotid plaque between T2DM patients with normoalbuminuria and microalbuminuria[45]. Therefore, increased albuminuria may be an independent risk factor for atherosclerotic lesions in T2DM subjects.

On the other hand, current results on the association between eGFR and atherosclerosis are also inconclusive in T2DM patients[22, 27, 40, 46, 47]. A related study reported that a lower eGFR was associated with a higher prevalence of atherosclerotic plaque in patients with chronic kidney disease [48]. Further studies found that there were no differences in CIMT values among different albuminuria groups, but eGFR was negatively correlated with CIMT values in T2DM patients[46, 49]. However, Yoon et al. revealed that UACR, rather than eGFR, was an independent risk factor for the grade of carotid atherosclerotic plaque in patients with T2DM[17]. Likewise, a recent study supported that T2DM, a common risk factor for atherosclerosis, attenuates the correlation between eGFR and carotid atherosclerosis [47]. Consistently, we also did not observe an association between eGFR and atherosclerosis in T2DM patients in the present study. Even though several factors including glucose-lowering medications which may influence insulin sensitivity were related to low eGFR levels, the correlation between eGFR and atherosclerosis disappeared after correction for these factors, which suggested that eGFR was not a true independent risk factor for atherosclerosis. The findings that FCP increased with decreased eGFR and increased UAE might be related to differences in C-peptide secretion and altered C-peptide metabolism in renal tissues as a major metabolic pathway[50]. Consequently, we thought that albuminuria but not eGFR was obviously associated with an increased risk of atherosclerotic lesions in T2DM patients.

Additionally, the present study further analyzed the comparison of atherosclerotic lesions in normal, normal eGFR and high UAE, low eGFR and normal UAE, and combined low eGFR and high UAE groups, which suggested that the coexistence of high UAE and low eGFR was closely associated with the deterioration of atherosclerotic lesions. This was consistent with the Atherosclerosis Risk in Communities (ARIC) Study which showed a 1.36-fold risk of CVD in patients with both moderately increased albuminuria and mildly decreased eGFR (UACR 30-299 mg/g and eGFR 60-89 ml/min/1.73 m^2^) compared with normal eGFR and albuminuria [33, 51].

There are several possible explanations for this result. One possible reason was that albuminuria was thought to reflect the systemic disease process, not merely a sign of impaired renal function [43]. The presence of albuminuria was linked to oxidative stress and vascular endothelial dysfunction [27, 29, 52], which play important roles in atherosclerosis. Another possible reason was that high UAE was accompanied by more conventional atherosclerotic risk factors. For example, patients with higher UAE exhibited higher SBP, DBP, TC, and TG levels in this study. Furthermore, to some extent, albuminuria potentially worsens atherosclerosis by increasing vascular stiffness, a constant indicator for hypertension[21, 53]. Moreover, albuminuria was associated with hyperlipidemia and vascular lipoprotein deposition, which promote the development and progression of atherosclerosis [38, 54, 55].

The strength of this study lies in the relatively large sample size. Thus, the findings were a true and accurate reflection of the relationship between albuminuria/renal function and atherosclerosis. Additionally, given that carotid ultrasonography alone may underestimate the extent of atherosclerosis in DM patients according to our previous findings [7, 56], we used carotid and lower extremity artery ultrasonography simultaneously to assess atherosclerosis, which was more comprehensive in addressing the features of atherosclerotic lesions. To the best of our knowledge, few studies have conducted ultrasonography in both the carotid and femoral regions [38, 57].

There were some limitations in the present study. As a cross-sectional study, it could not determine the cause-and-effect relationship between UAE and atherosclerosis in T2DM patients. Previous prospective studies have observed that albuminuria has a favorable prognostic effect on cardiovascular morbidity and mortality in patients with T2DM [58, 59]. Interestingly, this effect is particularly pronounced when GFR is normal or near normal [56]. The Nephropathy in Diabetes type 2 (NID-2) study showed that mortality and major cardiovascular events(MACE) could be significantly reduced with long durability through a multifactorial intervention in albuminuric T2DM subjects without previous MACE [60]. However, there is a lack of long-term follow-up studies on the association of UAE and eGFR with atherosclerosis. If the association of albuminuria with atherosclerosis is confirmed in prospective studies, early screening and intervention for diabetic macrovascular diseases should be undertaken to reduce the risk of future cardiovascular events in T2DM patients with elevated albuminuria. In addition, our study may suffer selection bias due to it being a single-center study. Thus, further prospective studies with more patients from multiple centers are necessary to verify the influence of albuminuria and low eGFR on atherosclerosis in T2DM patients. Furthermore, the use of metformin, insulin sensitizers, and IIA affected the assessment of insulin resistance, and HOMA2-IR was corrected to assess the correlation between UAE, eGFR, and atherosclerosis. Actually, a variety of factors might affect insulin resistance, and even if HOMA2-IR was corrected, it was still not possible to exclude all factors affecting insulin resistance, which in turn may affect atherosclerosis.

## Conclusions

In conclusion, high UAE, but not low eGFR, increases the risk of atherosclerosis in T2DM patients. Albuminuria is an independent risk factor for carotid and femoral atherosclerotic lesions. Therefore, there is a need to screen for diabetic macroangiopathy and to develop effective preventive measures against such cases, even in T2DM patients with slightly elevated albuminuria.

## Data Availability

The datasets used and/or analyzed during the current study are available from the corresponding author on reasonable request.

## Abbreviations

UAE: Urinary albumin excretion
eGFR: Estimated glomerular filtration rate
T2DM: Type 2 diabetes mellitus
CVD: Cardiovascular disease
IMT: Intima-media thickness
PAD: Peripheral arterial disease
BMI: Body mass index
WHR: Waist-to-hip ratio
CIMT: Carotid intima-media thickness
FIMT: Femoral intima-media thickness
DD: Duration of diabetes
AHA: Antihypertensive agents
LLD: Lipid-lowering drugs
APA: Anti-platelet agents
IIA: Insulin or insulin analogue
SBP: Systolic blood pressure
DBP: Diastolic blood pressure
WC: Waist circumference
ALT: Alanine transaminase
TC: Total cholesterol
TG: Total triglycerides
HDL-C: High-density lipoprotein cholesterol
LDL-C: Low-density lipoprotein cholesterol
FPG: Fasting plasma glucose
2-h PPG: 2-h postprandial plasma glucose
SUA: Serum uric acid
HbA1c: Glycated hemoglobin A1c
FCP: Fasting C-peptide
2-h PCP: 2-h postprandial C-peptide
HOMA2-IR: HOMA of insulin resistance
